# “You Obviously Just Have to Put on a Brave Face”: A Qualitative Study of the Experiences and Coping Styles of Men With Rheumatoid Arthritis

**DOI:** 10.1002/acr.22951

**Published:** 2017-02-25

**Authors:** Caroline A. Flurey, Sarah Hewlett, Karen Rodham, Alan White, Robert Noddings, John R. Kirwan

**Affiliations:** ^1^ University of West EnglandBristolUK; ^2^ Staffordshire University, Stoke‐on‐KentStaffordshireUK; ^3^ Leeds Beckett UniversityLeedsUK; ^4^ University Hospitals Bristol NHS TrustBristolUK; ^5^ University of BristolBristolUK

## Abstract

**Objective:**

To explore the experiences, coping styles, and support preferences of male rheumatoid arthritis (RA) patients.

**Methods:**

Six focus groups comprised 22 men with RA. Transcripts were analyzed using inductive thematic analysis.

**Results:**

Three overarching themes describe the experiences, coping styles, and support preferences of men with RA. In “challenges to masculinity,” the men described a “reduction in strength and abilities,” which can lead to loss of independence, “challenges to masculine identity and role,” and “loss of power and control.” Coping by “getting through life with RA” meant dealing with RA by “just getting on with it,” “information seeking,” engaging in “destructive behaviors,” and “withdrawing socially.” Preferred “sources of support” tended not to include friends, as they were perceived to lack understanding or support. For acceptable support the men reported a preference for information‐giving sessions rather than a discussion group, but there was no agreement on whether these should be mixed‐sex or men only, or who should run the sessions.

**Conclusion:**

Male patients reported a range of coping styles and support preferences to address their experiences of living with RA, many of which may not be shared with women. Further research is needed to investigate whether these findings exist in a larger sample and whether the support preferences of men with RA are broadly different from those of women with RA to decide whether there is a clinical need to design a service for the potentially different needs of men.

## INTRODUCTION

Rheumatoid arthritis (RA) is a chronic, progressive, and systemic autoimmune disease, characterized by fluctuating symptoms such as pain and fatigue with associated emotional, social, financial, and societal burden 1, 2. RA affects more women than men, with approximately 30% of the RA population being male 3, 4, and RA may take a different course in women compared to men 5, 6.

Box 1 **Significance & Innovations**

- Men with rheumatoid arthritis (RA) report a challenge to their masculine identity due to the impact of RA. They therefore employ strategies to hide their RA in public in order to maintain their masculine image.
- Some men use destructive behaviors to cope with their RA, potentially causing more pain and joint damage.
- Many men reported not talking to their friends about their RA due to a perceived lack of understanding and support.
- Men with RA report being reluctant to discuss emotional issues with their rheumatology team unless explicitly asked with a direct question.

Evidence from several diseases indicates that long‐term conditions impact differently on men compared to women 7, 8, suggesting that men need their own health strategy 9. However, there is a dearth of literature exploring the impact of RA on men and their self‐management strategies. A comprehensive literature review 10 found no consensus on whether sex affects ability to cope with RA. However, findings did suggest that men use fewer and less diverse coping strategies than women 11, 12. If men do cope differently than women, it is likely they would have different support needs to suit these different coping styles. It is therefore of note that current self‐management interventions in RA have been designed and tested mainly in women, with randomized controlled trials of RA self‐management interventions reflecting the preponderance of women with the condition 13, 14, 15, 16.

Despite these indications that men may experience and deal with RA differently than women, only 2 studies (both qualitative) have focused solely on men with RA. One 17 recruited patients from only 1 UK hospital and did not explore coping strategies. The other 18 was conducted with US war veterans, who may have very different experiences of life and therefore have developed different coping strategies than the general male population. Neither of these studies explored the support preferences of men.

While research in other conditions suggests that men need their own health strategy, this has not been sufficiently investigated in RA. As a first step it is necessary to qualitatively explore and understand in greater depth how men experience and cope with their RA and to decide whether this is an issue worthy of further investigation in a larger sample of patients. Therefore, the current study aimed to explore the experiences of male RA patients and the impact RA has on their lives, how they are currently coping with and managing their RA, and whether they would like a support intervention, and if so, then their preferred delivery style and broad content.

## PATIENTS AND METHODS

#### Patients and focus groups

Male patients with clinician‐diagnosed RA 19, 20, 21 were invited to participate in focus groups by the researcher (CAF) or local research nurse. Every man attending a rheumatology outpatient appointment at 1 of 3 UK hospitals on the days of recruitment was invited to take part.

A topic guide (Table 1) was developed based on a literature review and discussions with the study team, including a male patient research partner (RN), and was used to facilitate discussion in the focus groups. These followed an iterative process 22, with new concepts emerging during data analysis being explored in subsequent focus groups. A prestudy questionnaire captured demographic data and disability (Health Assessment Questionnaire [HAQ]) 23 (Table 2). The focus groups were conducted by the first author (CAF) with a subset cofacilitated by a patient research partner (RN). As both a man and a person with RA (the lead researcher is neither), the patient research partner brought a different perspective and insight to the focus groups to inform followup questions 24. The focus groups lasted approximately 2 hours, were digitally recorded, and transcribed verbatim. Ethics approval was granted by the London‐Bromley Research Ethics Committee (reference 13/LO/0852) and written informed consent was obtained from each participant before each focus group.

**Table 1:** Topic guide used to facilitate focus group discussions

What is it like to have rheumatoid arthritis (RA)?
What is a good day with RA?
What is a bad day with RA?
What do you do to manage your symptoms?
Do you have to ask other people to do things for you?
What do you miss doing since being diagnosed with RA?
What changes have you made to your life?
Have you given up any activities due to being diagnosed with RA?
Have you ever been angry because of your RA?
What do you do to manage your anger/emotions?
Do you talk to anyone about your RA? Who?/Who do you see as your main support?
Have your expectations/ambitions in life changed?
What do you think of the care you receive from rheumatology?
Would you like to change anything about the care or support you receive?
What do you think is important to include in an RA self‐management/information program?
Who do you think should deliver this type of group (e.g., male/female)?
Where do you think the group should be delivered (at the hospital/elsewhere)?
Do you think women deal with RA differently than men? (if so how?)
Do you see a difference between men and women in the waiting room?
Is there anything positive about being a man with RA? (what?)
General followup questions:
What do you think about that? (use think, not feel)
Do you think that's different for women?

**Table 2:** Individual participants' demographic and disease‐related data (n = 22)^a^

Pseudonym	Focus group	Age, years	Disease duration, years	HAQ	Employment status^b^	Marital status	Current medication
David	A	58	19	0	Full time	Divorced	DMARDs
Robert	A	60	12	2	URA	Married	DMARDs
James	A	72	0.7	0	Retired	Widowed	DMARDs, steroids
Richard	B	64	14	1.875	Retired	Married	DMARDS, biologic agents
John	B	61	5	2.375	URA	Married	DMARDs
Charles	B	69	2	0.75	Retired	NK	DMARDs
Will	B	61	1	1.125	Full time	LWP	DMARDs
Mike	B	60	12	2.375	URA	Divorced	DMARDS, biologic agents
Mark	C	49	5	0.125	Full time	Married	DMARDS, biologic agents
Tom	C	66	NK	1.25	Retired	Married	NK
Paul	C	57	19	2.25	URA	Married	DMARDS, biologic agents
George	C	74	9	1.25	Retired	Married	DMARDs
Brian	D	63	8	0.5	Full time	Single	DMARDs
Ian	D	64	0.2	1.25	Part time	Married	DMARDs
Steve	D	44	1.5	1.25	Full time	Divorced	DMARDs
Henry	D	71	6	0	Retired	Divorced	Steroids
Alan	D	64	11	2.625	Retired	Married	Biologic agents
Ron	E	60	17	1.25	URA	Married	Biologic agents
Edward	E	75	10	1.875	Retired	Single	DMARDs
Frank	E	75	17	1.5	Retired	Married	Biologic agents
Albert	F	75	8	NK	Retired	Married	DMARDs
Fred	F	64	10	NK	Retired	Divorced	None (refused medication)
Mean		63.9	8.9	–			
SD		8.1	6.1	–			
Median		–	–	1.25			
IQR		–	–	0.69–1.91			
Range		44–75	0.2–19	0–2.375			

a HAQ = Health Assessment Questionnaire (0–3, where 3 = severe disability); DMARDs = disease‐modifying antirheumatic drugs; URA = unemployed due to rheumatoid arthritis; NK = not known; LWP = living with partner.

b Option of unemployed (other) was given but not selected.

#### Statistical analysis

Data were analyzed using inductive thematic analysis, a method for identifying, analyzing, and reporting patterns (themes) within data without trying to fit it into a preexisting coding frame, or the researcher's preconceptions 25. Data were analyzed according to Braun and Clarke's guidelines 25 and managed using NVivo 8 26. The first author (CAF) analyzed all the transcripts, and a sample was independently analyzed 27, 28 by 3 researchers (SH, KR, and AW) and a patient research partner (RN). Team discussions and comparison showed that they reached comparable conclusions to the first author (CAF).

## RESULTS

**Figure 1:**
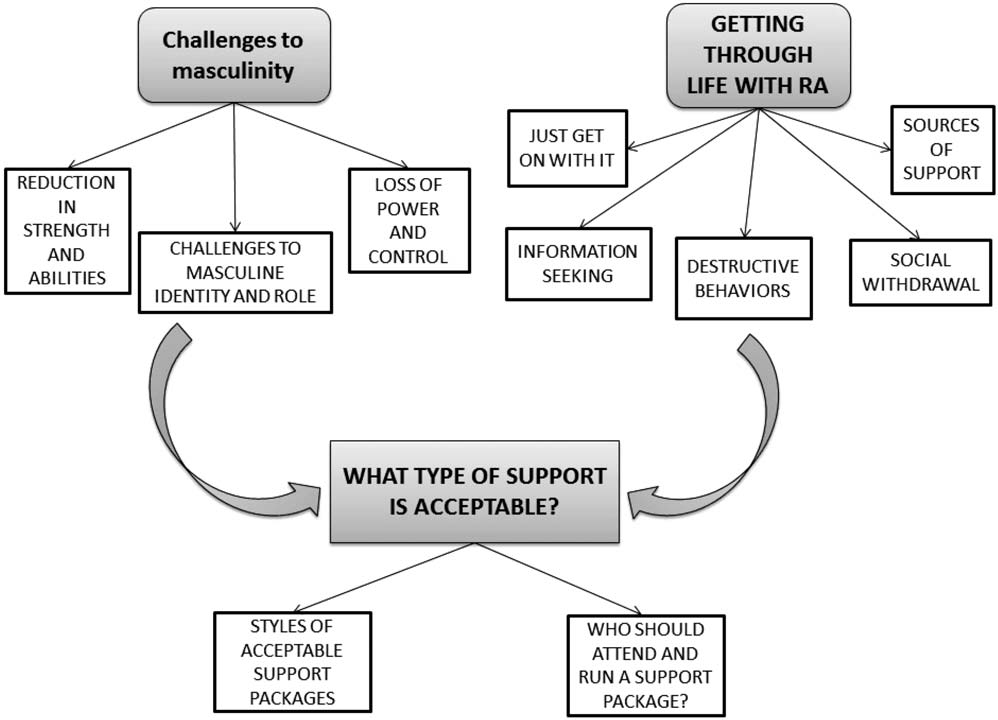
Thematic diagram of men's experiences of living with and managing rheumatoid arthritis (RA).

Twenty‐two men with RA participated in the study with a median (interquartile range [IQR]) age of 64 years (60–70.5 years), range 44–75 years, which is slightly older than the RA population 29. Median (IQR) disease duration was 9.5 years (5–12 years), range 2 months to 19 years; HAQ score was 1.25 (0.69–1.90), range 0–2.375 (Table 2); and all participants were white and British. Six focus groups (A–F) were conducted. Small focus groups of 4 to 6 participants were planned to allow the men to feel comfortable discussing thoughts and feelings openly 30. However, due to nonattendance some focus groups were smaller than planned (2 to 5 participants in each). Thematic analysis identified 3 overarching but interconnected themes relating to the way men experience and manage their RA, and the support they require (Figure 1).

#### Challenges to masculinity: “it's not a very macho thing.”

Participants described the various challenges to masculinity they experience due to their RA and how they dealt with this.

##### Reduction in strength and abilities

A reduction in strength and the ability to do things previously taken for granted was raised as an important issue. For some this reduction in strength alters their ability to do their job as they used to: “Some of these radiators I have to lift up are so heavy. Years ago I'd just sling them on my shoulder and walk about. Not anymore” (Mark). Participants explained that this reduction in strength and ability leads to the need to ask for help, which some men view as a challenge to their masculinity: “It puts you in a position where you have to ask for help and it's not a very sort of macho thing” (Will). This is particularly apparent when they need to ask for help with physical tasks from their wives or partners, causing them to challenge their assumption that men are physically stronger than women: “It is horrible, you've got to say to your wife, ‘I can't undo this bottle,’ and she just goes like that and it's undone” (Alan). Some participants explained that it is more acceptable to pay a tradesman to do a job they need help with rather than accept a favor: “I find it easier to get a stranger in than a friend of the family, a lot easier, personally. I've got a grandson, he's a carpenter, anything we need to do he'll come and do. Then you'll have an argument with him because he won't take any money and I find that embarrassing. So I don't ask him anymore” (Frank).

Reduction in abilities also leads to a loss of independence, and the emotional and physical impact of this resonated with all participants: “One of the worst things you can do, when you've got it bad, is get down on your knees. Cause you can't get up…it's so soul destroying because what it does, it takes away your…” (James). “…Yeah, your dignity” (Robert). Some participants discussed the loss of financial independence due to a reduction in work: “Generally, financially and all that sort of thing, you lose your independence and that's what I found now I've stopped, earning extra money” (Edward).

##### Challenges to masculine identity and role

The impact of RA on valued roles and self‐image can challenge masculine identity. These men's altered self‐image seems to conflict with their idea of themselves as a healthy man: “When I first got it…it was quite a shock…I thought initially, why me? Because I've always been active” (Charles). To protect their masculine identity in public these participants often made attempts to conceal their RA: “You go to shake their hands, and I don't know what it is about certain guys but they've really got to grip you, and you obviously just have to put on a brave face, because it really hurts” (Mark). The dominant discourse in these discussions was around gendered roles, and the loss of ability to carry out the “man's tasks” within the home was felt by many of the participants: “As a man you should be the main person doing the lawns, the gardens, round the house” (Richard). Another important part of masculine identity is the man's role as the breadwinner, and work was therefore raised as an important issue: “When we were kids we were brought up that a fella goes to work, a wife stays at home” (Mike). “I couldn't do my job anymore so they laid me off like, made me redundant and that hurt” (Frank). Some men also felt their role in the family was compromised: “I used to play fight with him [son] and everything, pick him up and chuck him up the garden and stuff like that and I just can't anymore…Sons look at dads as being just like, ‘Dad, you're invincible,’ and then all of sudden you can't do it” (Steve).

##### Loss of power and control

Underlying the challenges to strength, ability, independence, and identity is the loss of power and control, which can lead to feelings of helplessness. This lack of control due to a body that no longer behaves as expected or desired can extend to other areas of life, causing anger and frustration: “Well I can't pick that box up guvnor.” “Well, you ain't working for me,” sort of thing, you know what I mean” (Paul). “That makes you angry” (Tom). “Yeah, angry ain't the word for it” (Paul).

A further loss of power and control for some participants was through power imbalance during interactions with health care professionals. This was particularly felt when they did not feel believed or treated with care, which could leave them feeling helpless: “They know that men don't go [to the doctor] unless they've really got to, and even then they'll still fob you off” (Robert). “I had a similar problem… I was leaning against the x‐ray plate, and this nurse…” (Richard). “Tried to yank your arm behind your back” (John). “…just dragged my arms back and pulled…It was so painful” (Richard).

The men appreciated efforts by health care professionals to reduce this power imbalance, and seemed to value the approach of a clinician that reinforced their masculine status: “He's [rheumatologist] really down at your level isn't he, he's a man's man…he's just a bloke” (Ron).

#### Getting through life with RA: “Just get on with it.”

Participants found different ways of dealing with their RA, which could be both proactive and passive.

##### Just get on with it

Participants discussed the need to just get on with things rather than complaining about their RA: “I just accept it and get on with it, what else do you do?” (Ian). “Yeah, exactly. My last visit to the rheumatologist, his actual words to me were I'm too stoic” (Henry).

They discussed practical self‐management strategies such as pacing and planning, and acknowledged the importance of keeping active: “You just pace yourself” (Robert). “Pace yourself and have a little rest between things” (James).

Participants also discussed using tools to help them manage. The tools that were talked about were often those used to make masculine tasks (such as do‐it‐yourself) easier rather than for disability, such as walking aids: “You get a little electric thingy for screws [electric screwdriver]” (Robert). Some men explained that they make their own devices or disability aids to help with their RA: “It helps if you are a bit that way, that you can make things to help yourself” (David). “I've actually made the sofa about that much higher, put extra legs on it” (Robert).

##### Information‐seeking

In an attempt to retain control of their RA, these male patients sought information to keep themselves informed. This came from many sources, including journals, newspapers. and the internet: “I saw that in The Mail” (Tom). “This was in the Lancet” (George). They valued medical intervention, and the discourse in the focus groups often centered around medication, with all the men knowing their medication regimen in detail: “What dose of methotrexate are you on?” (Richard). “15 mg subcut, that's injected” (Will). They also valued regular monitoring by their medical team, which provided current information about their condition: “I go for monthly checks, so you know you're being monitored, you can't ask for more than that” (George).

##### Destructive behaviors

In an attempt to get on with their lives despite their RA, some men discussed pushing themselves to their limits, while disregarding any consequences: “I will work all day and then wonder the next day why I can't use my hands…and my knees have had it” (Edward). “…I feel you've got to finish the task” (Ron). Some men reported using exercise as a way to vent frustration and anger. This was sometimes at the expense of putting strain on the body and joints: “I use [running] as a way of getting rid of the frustration and the anger; I just run and run and run until I can't run anymore…I just feel like just punishing myself. I know I shouldn't because I know I'm going to pay for it in the long run” (Steve). Risk‐taking behaviors were also discussed, such as skipping medication to accommodate their social life and drinking more alcohol than recommended with their medication: “If I'm going out, like the weekend, I don't take one methotrexate that week. I know slap on the wrist there” (Steve). “[Rheumatologist] said you can drink, as long as it's within moderation…well, I'm going on holiday in 6 weeks’ time. I mean, what do you go on holiday for? To get drunk and fall down” (Will).

##### Social withdrawal

Some men reported dealing with the emotional aspects of their RA by avoiding people: “I live on my own…so I can rattle around, chuck stuff and just be thoroughly frustrated and just lay in bed all day” (Steve). Social involvement could also be withdrawn due to no longer feeling able to join in with activities. Some men appeared black and white in their thinking and were therefore unable to consider ways to replace lost activities: “I've lost my social life as it is now, because I don't go out playing pool, I don't go out playing golf, which are the two main things I done” (John). “…I gave up going to pubs completely because I just didn't feel right going into a pub and having soft drinks” (Mike).

##### Sources of support

Many of the men reported receiving support from wives and partners, and some were supported by other family members such as adult children: “Luckily I've got a very understanding wife” (Frank). “I talk to my daughter sometimes about it…she seems more understanding than anyone else” (Alan). While some participants felt able to talk to health care professionals about the emotional impact of RA, others found this difficult: “I've always been able to speak to [nurse] about” (Alan). “I wouldn't want to discuss it with [rheumatologist], not interested, no” (Paul). Some men indicated they would need to be explicitly asked about emotional impact to enable them to discuss it: “No they didn't [discuss support options], on the other hand I didn't tell them I was depressed either…I went and seen [rheumatologist], we talked and we came out” (Frank). “I think when you're in the state you're in with this and you know, it depends on the questions what's being asked” (George).

However, the rheumatology team may not always explicitly ask about emotional well‐being, and less direct questions are not interpreted by the men to include emotional impact: “They ask how I've been and how I am being, I tend to take that from an RA perspective rather than an overall perspective…they never split the question” (Richard).

The majority of participants reported that their friends are not understanding and they would be unlikely to discuss their RA with them: “I go [clay] shooting…if I complain just for a minute…they'll say ‘stop bloody moaning.’ That's all you get” (James). “No, they're not understanding at all, no, not unless they've got something wrong with them” (Mark). “That's why we all keep schtum” (Paul).

#### What type of support is acceptable? “We'd end up just moaning.”

##### Styles of acceptable support packages

Many participants agreed that a group session would be useful, but it was important that the group had a purpose. They emphasized their lack of interest in a discussion about their condition, as this was seen as unhelpful and something women do: “Just speaking to other people with the same complaint, I can't see what good that would do. Cause all we'd end up is just moaning at one another” (Frank). “Like a gaggle of women and they're cracking their jaws and…sometimes they talk a lot and say nothing” (Fred).

Despite these views, many participants seemed to value the opportunity to talk to other men with RA in the research focus groups: “They [other people] just don't understand at all how it gets you, whereas you can recognize everything that everybody [in the focus group] is saying because you've been through it” (George). “I've never done it [talked to other men with RA] before” (David).

However, when asked if they would come to a similar group as a support service, the majority emphasized that they had attended for the purpose of research and not to help themselves: “I don't know what I'd say [in a support group]. I'm here to help you [researcher]” (Frank).

Ideas for group sessions were information giving, or a question and answer session: “Something coming the other way in relation to what research has been done and what the findings are” (George). “Say for instance, every two months, a consultant is in a room and maybe there's twenty people could all discuss what's going on” (John).

##### Who should attend and run a support package?

The men had different views on whether the group should be mixed or single sex. Some preferred an all‐male group due to potential difficulties opening up with women present, while others were interested in hearing women's views: “I went to one [support group] and it was a mixed group, and I didn't say anything, sat there and didn't say anything…There were quite a few women there, they were talking and I thought I don't want any of this” (Frank). “I'd like to hear how [women] cope with it and how theirs differs and how they feel compared to how we feel” (Steve).

In terms of who should run the group, some patients valued the knowledge of medical professionals, while others would prefer an expert patient helping to run the group: “Medical people I would say because they could answer questions if you have them” (Mark). “Staff led but patient run…the staff should sort of stand back, or be there for reference, and let the patients get on with talking, because they are the ones who are actually experiencing it” (Will).

## DISCUSSION

These male RA patients reported the impact of RA on their life through reduction in strength and ability, challenges to masculine identity and role, and loss of power and control. They dealt with these issues by just getting on with things, information seeking, concealing their RA in public, engaging in destructive behaviors, and withdrawing socially. The majority of participants would not discuss their RA with friends due to a perceived lack of understanding or support. Acceptable support was suggested as an information‐giving session rather than a group discussion. This raises the question as to whether these issues are pertinent to all (or most) men with RA and whether they are more common in men than women with RA, as this would have important implications for the design of support services.

Loss of independence and increased need to ask for help has been identified in previous qualitative studies with predominantly female patients 31. However, participants in the current study reported asking for help as a direct challenge to their masculinity. The need to incorporate RA into identity has been identified in previous RA research with both men 17 and women 31. However, the men in the current study were reluctant to incorporate RA into their public identity and reported employing strategies to conceal their RA in public. This may be an attempt to “pass” as fully and self‐evidently masculine for either a real or imaginary audience 32.

The coping strategies reported by the men in this study to deal with the physical and emotional impacts of RA support the idea that men try to behave according to traditional (American and British) ideals of stoicism and emotional self‐sufficiency 33, 34, 35. These male patients reported being willing to talk to their rheumatology team if directly asked about their emotional well‐being, which may be because they value emotional support, but feel uncomfortable acknowledging this 36. Thus, rather than asking open‐ended questions (e.g., “How are you doing?”), clinicians should be encouraged to explicitly ask men about psychological and emotional issues. There is also value in taking an indirect approach, such as talking about “safe issues” like work or family, as opposed to direct questioning about feelings 37. Patients reported a perceived lack of support from friends, supporting the proposal that men have poorer social capital than women 38, 39.

A comprehensive literature review of the acceptability of different types of support for men with long‐term conditions 36 found that men need support interventions to have a purpose, be structured, and provide opportunities to gather new information 35, 40. The men in the present study report preferring information sessions to group discussions. This supports the theory that while women prefer face‐to‐face conversation, many men find this too personal and instead benefit from “covert intimacy” 41, which tends to take the form of shoulder‐to‐shoulder conversations, while engaging in shared activities such as the Men's Sheds project 42. The men participating in this study emphasized that they were attending the focus groups to help with the research project rather than to help themselves. This supports previous findings that suggest men may be more likely to attend support services as a volunteer 42.

This study may have limitations because the whole sample was white and British and the majority were over age 55 years. However, the opinions of relatively younger men from the age range with RA were captured in this sample, and due to the nature of qualitative research a separate study would be necessary to fully capture the potentially different experiences of men with RA from different ethnic backgrounds. It is possible that this study is missing the voices of men who were unable to take part in scheduled focus groups (e.g., due to work commitments), or the “strong, silent” men who may be reluctant to participate in qualitative research 43. Focus groups can be criticized for producing consensus opinion or favoring the most dominant members of the group 44. However, they were chosen due to the potential for group discussion to elicit ideas (e.g., suggestions for support provision) that may not arise from one‐to‐one interviews 45. Further, focus groups were useful in identifying whether the men valued the opportunity to talk to other men with the same condition. A further limitation of this study is the small size of some of the focus groups. In an attempt to keep the groups of an acceptable size for men (n = 4–6) 30, we avoided overrecruitment, which may have increased the likelihood of low attendance 46. Strengths of the study are that it sampled patients for a range of disease duration and disability and from 7 consultants across 3 UK hospitals, thereby accessing a range of disease experiences and care pathways. Further, a patient research partner contributed to the study design, data collection, and interpretation (RN).

These novel data focusing on men with RA suggest that men have a range of experiences living with RA and a range of coping styles to address the issues they experience. The similarities between the issues identified as specific to men with other long‐term conditions suggest that some of these experiences, coping styles, and support preferences may not be shared with women. Thus, there is a realistic possibility that many men with RA are not being served by current self‐management interventions. Further research should investigate the generalizability of these findings and whether the coping styles and support preferences of men with RA are broadly different than those of women with RA. This is now being addressed through a survey study informed by these findings, which will enable an informed judgement of whether there is a clinical need to provide services tailored toward the potentially different needs of men.

## AUTHOR CONTRIBUTIONS

All authors were involved in drafting the article or revising it critically for important intellectual content, and all authors approved the final version to be submitted for publication. Dr. Flurey had full access to all of the data in the study and takes responsibility for the integrity of the data and the accuracy of the data analysis.

### Study conception and design

Flurey, Hewlett, Rodham, White, Noddings, Kirwan.

### Acquisition of data

Flurey, Noddings.

### Analysis and interpretation of data

Flurey, Hewlett, Rodham, White, Noddings, Kirwan.
